# Effect of Chair Yoga Therapy on Functional Fitness and Daily Life Activities among Older Female Adults with Knee Osteoarthritis in Taiwan: A Quasi-Experimental Study

**DOI:** 10.3390/healthcare11071024

**Published:** 2023-04-03

**Authors:** Ching-Teng Yao, Bih-O Lee, Hong Hong, Yi-Ching Su

**Affiliations:** 1 Master Program of Long-Term Care in Aging, Kaohsiung Medical University, Kaohsiung 80708, Taiwan; 2 Center for Innovative Research on Aging Society, National Chung Cheng University, Chiayi 62102, Taiwan; 3 College of Nursing, Kaohsiung Medical University, Kaohsiung 80708, Taiwan; biholee@kmu.edu.tw (B.-O.L.);; 4 Graduate Institute of Adult Education, National Kaohsiung Normal University, Kaohsiung 80201, Taiwan

**Keywords:** effect, chair yoga therapy, knee osteoarthritis, functional fitness, daily life activities

## Abstract

This study aims to examine the effectiveness of chair yoga therapy on improving functional status and daily life activity scores in older female adults with knee osteoarthritis living in the community. A quasi-experimental design was adopted. In total, 85 female participants with knee osteoarthritis were assigned to the chair yoga therapy intervention group (*n* = 43) or the comparison (*n* = 42) group. A 12-week chair yoga exercise program was provided to the intervention group two times per week from January to April 2020. The primary outcomes, which include changes in physical functional ability, body mass index, and biophysiological indicators, were evaluated for all participants in the pre- and post-measures time periods. The analysis shows that the participants had a significantly higher level of functional fitness and daily life activity scores after the chair yoga intervention. This finding indicates that the chair yoga program was effective in improving the functional fitness and daily life activity scores of community-dwelling elderly females with knee osteoarthritis.

## 1. Introduction

The rapid growth of the geriatric population is a prevalent phenomenon worldwide [1]. Aging accelerates the deterioration of physiological and psychological health, resulting in increased occurrences of falls, morbidity, depression, neuro-related diseases, and mortality [2]. These age-related diseases lead to dependence, disability, and a poor quality of life, which worsen the severity and complexity of diseases. Recently, osteoarthritis has rapidly increased due to aging populations, with a worldwide prevalence of 22.9% among individuals aged 40 years or over [3]. In Taiwan, the current prevalence of osteoarthritis among the older population is about 37% in individuals over 50 years old [4]. Among degenerative diseases, osteoarthritis is the major cause of long-term disability among older adults in Taiwan, affecting 3.45 million people [5]. The etiology of osteoarthritis consists of aging, occupational injury, chronic inflammation, a history of trauma, hereditary factors, and obesity, with approximately one-third of the elderly population suffering from injury, pain, and discomfort [6]. Women have a significantly higher risk of osteoarthritis than men, especially in the postmenopausal period. Pains in the knee and hip joints, namely, knee osteoarthritis and hip fracture, are the chief complaints from osteoarthritis patients. In addition, osteoarthritis is accompanied by pain, stiffness, and reduced physical functions, leading to limitations in walking capacity and physical activity, as well as decrements in the activities of daily living (ADL) [7,8,9]. The restrictive capacity of knee osteoarthritis increases the risk of geriatric symptoms and disease, including falling, emotional problems, lower quality of life, and psychosocial distress [10,11].

Exercise is a core nonpharmacological therapy approach for managing osteoarthritis problems, decreasing stiffness, and increasing joint stability and mobility. The effects of exercise improve functional ability in knee osteoarthritis. Participating in isokinetic and eccentric contraction exercise programs, land-based exercises, and flexibility exercises is recommended for training knee osteoarthritis, with benefits such as strengthening the muscles around the knee, including the quadriceps, lateral knee muscle, and hamstring muscle [12,13]. Yoga practice builds muscle strength, improves joint flexibility, and increases the degree of motion, strengthening muscles, imparting flexibility, and improving physical balance [14]. Regular exercise has numerous mental health benefits for older adults [15]. Numerous evidence-based randomized home-visit trials have shown the benefits of exercise interventions, such as aerobic, resistance, or mind–body exercise [16]. The effectiveness of multiple-fitness training reduces the risk of frailty, disability, and morbidity in all ages [10,17,18]. Furthermore, low-impact exercise has positive effects on reducing pain and tenderness from knee osteoarthritis as well as improving physical function and range of motion [19].

Yoga is a low-impact and moderate-intensity exercise characterized by its slowness, training of core muscles, flexibility, physical coordination, non-competitiveness, and ease of access; therefore, yoga is highly recommended as an exercise program for the elderly to improve their fitness [9,18,20]. Previous studies have demonstrated the positive relationship between regular engagement with yoga and health outcomes in terms of reducing blood pressure and fall-related injury, enhancing mobility, and reducing depression and stress. Moonaz et al. suggested that yoga is a better type of exercise for older people with arthritic hips, improving muscle strength, joint flexibility, and physical balance compared with a comparison group [21]. Nevertheless, the reported effects of yoga on knee osteoarthritis are inconsistent. Traditional yoga is similar to static stretching, which includes spinal/leg flexion and rotation while standing or lying on the floor, and those poses may discourage the elderly from participating in the relatively risky exercise [22,23]. However, yoga is not merely a form of exercise that strengthens muscles, imparts flexibility, or improves physical balance; it also positively affects the mental and spiritual domains of health. The provision of regular yoga exercise in community settings can improve the flexibility and physical coordination of older adults with osteoarthritis. Chair yoga exercise, supported by chair equipment, provides a relatively safe and stable environment for the elderly with osteoarthritis to enhance their muscle endurance and flexibility compared with yoga in a standing pose [17,22,24].

Findings from two intervention studies have shown that chair yoga therapy can reduce depression and pain and increase life satisfaction in older adults with osteoarthritis [11,25]. However, not many chair yoga intervention studies have been performed to date. Knee osteoarthritis is common in older female adults, and it is regarded as a serious health issue for those with a previous history of falls or who are living without a caretaker [11,19,22,23,25,26]. For these reasons, we study chair yoga as a culturally acceptable method to develop a functional fitness and daily life activity improvement strategy for older female adults with osteoarthritis. An expressive chair yoga therapy intervention, which has not yet been tested in Taiwan, can be tailored to the needs, predispositions, and preferences of older female adults with knee osteoarthritis, improving their functional fitness and daily life activity scores. Therefore, the purpose of this study is to explore the effects of chair yoga therapy interventions on the functional fitness and daily life activity scores of older female adults with knee osteoarthritis in Taiwan.

## 2. Methods

### 2.1. Study Design and Procedure

Quasi-experimental research is conducted in southern Taiwan with a sample population of older female adults with knee osteoarthritis who are more than 65 years of age. Two communities were selected as study sites to recruit participants. To avoid bias, the two communities were assigned to either the comparison or intervention group using a random number generator. The recruitment activities took place in the two communities on the same day. Subsequently, the participants completed a form to consent to their involvement in the study. The inclusion criteria for this study were as follows: (a) woman aged 65 years old or above; (b) self-reported history of knee osteoarthritis diagnosed by physicians; (c) capable of mobility with assistive devices; (d) limited in participating in any kinds of standing exercise; and (e) currently not participating in chair yoga or an exercise program and has not participated in any other exercise research projects during past six months. Moreover, participants with the following conditions were excluded from the study: diagnosis of dementia, disabilities, or incapability of communication. Participants in the intervention group received additional structured chair yoga therapy sessions. The comparison group participated in regular activities in the community and received the same scale tests at the same time points as the intervention group. This study assessed and compared changes in the functional fitness scores of participants in each group using a senior functional fitness test at 12 weeks from the baseline. It also assessed the change in the IADL of participants at 12 weeks from the baseline.

Sample size calculation was based on the hypothesis to estimate the mean difference between the two independent groups (intervention and comparison) using an independent *t*-test. The inputs for sample size calculation (G*Power software) were effect size (d) = 0.8, two-sided alpha error = 0.05, power = 0.8, and allocation ratio = 1 [27]. The sample size in each group was 36 participants, with a total sample size of 72 study participants. Of the 89 potential subjects who were screened, 85 were enrolled and assigned to the intervention (n = 43) or comparison group (n = 42).

### 2.2. Research Instrument

A senior functional fitness test developed by Rikli and Jones (2013) was used to assess physical statuses [17]. The 7-item senior functional fitness test was administered through interviews and assessed independent lower- and upper-body strength, aerobic endurance, lower- and upper-body flexibility, and agility/dynamic balance. Two professional research assistants conducted the measurements. All tests were conducted with strict adherence to the senior fitness test instruction manual [28]. All measures were test–retested, and the best value for each test was recorded for analysis. The intraclass correlation coefficient values for all tests were higher than 0.98 in this study.

The structured questionnaire adopted in this study comprised demographic questions (e.g., age, education, and number of chronic diseases), and a Chinese version of the Lawton Instrumental Daily Living Scale (IADL) was used to assess eight primary and psychological functions [29]. The IADL scale is used to identify how an elderly subject carries out daily activities, recording improvements or deterioration over time [30]. It was originally designed to monitor prognosis and treatment in chronically ill elderly patients; however, it is now being used to evaluate the independent abilities of older adults in a wide range of settings. Its summary score ranges from 9 to 20 points, with a score of 20 meaning they are independent and a score of 9 meaning they need assistance or supervision in performing tasks. The Chinese version of the scale was reported to have established validity and reliability [31]. Cronbach’s alpha for the IADL used in this study was 0.89, demonstrating good internal consistency.

### 2.3. Chair Yoga Intervention Technique

**Figure 1:**
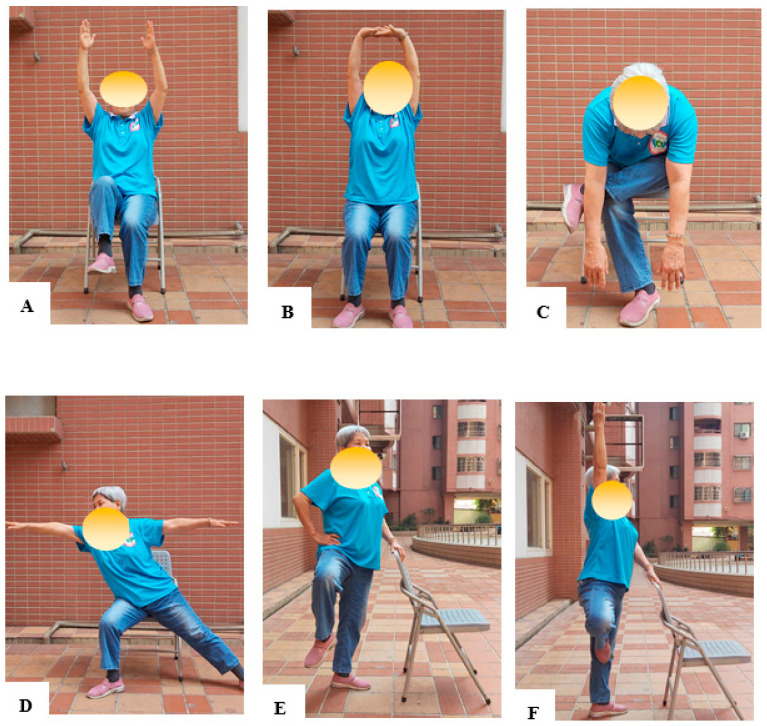
The various chair yoga positions performed by participants are shown in this figure. Some examples are (**A**) reaching both arms forward and waving them up and down, (**B**) seated mountain pose, (**C**) down dog with chair, (**D**) hero pose, (**E**) Lifting the knees while standing, (**F**) tree pose.

The chair yoga program was designed based on the results of a previous study [32]. The program development included a preliminary review and evaluated content validity using an index created by a panel of five geriatric experts in the fields of nursing, occupational therapy, geriatric medicine, and long-term care. Table 1 provides a brief explanation of the content of the chair yoga therapy activities. Trained yoga personnel from the institute conducted 110-min sessions of chair yoga intervention. Such sessions were held twice weekly for a duration of 12 weeks. Most of the yoga postures were completed in a sitting posture. This was followed by a few standing postures, and during the final posture, participants were again seated to rest and breathe. The chair yoga intervention focuses on balance and postures that ultimately improve confidence and build the physical functions of older adults in the community, such as muscle strength, flexibility, and balance capabilities. The classes emphasized breathing throughout all postures. Additionally, yoga postures were appropriately modified in case of disease-related complications or any other critical issue after discussion with the yoga instructor. Examples of chair yoga training are shown in Figure 1. The chair yoga program is demonstrated in Youtube videos [33].

### 2.4. Ethical Considerations

All participants provided written consent. No identifying personal data are included in this paper. This study was approved by the appropriate institutional review board (National Cheng Kung University, Taiwan). Approval for this study was obtained on 10 March 2020 via letter number NCKU-IRB-2020-207. All study-related documents along with a detailed protocol have been submitted to NCKU. Any changes in protocol and protocol deviations or violations were reported by investigators of NCKU.

### 2.5. Statistical Analysis

Data analysis was performed in SPSS software (version 24.0, IBM). The continuous variable was reported as mean ± standard deviation based on the distribution of data. The data normality test was carried out from the pre- and post-test functional fitness and daily life activity scores. The normality test value shows a normal distribution because the significance value (*p*) of the Kolmogorov–Smirnov test is >0.05. In this study, *t*-tests or an ANOVA analysis of variance were used for comparing continuous variables among or between subgroups. An ANOVA analysis of covariance was also used to compare functional fitness and daily life activity scores between the groups when scores differed significantly at baseline. Statistical significance was considered if the *p*-value was less than 0.05 at 95% confidence.

## 3. Results

### 3.1. Demographics of Participants

In this study, 89 participants met the criteria, and 4 participants withdrew from the program without any notice. Thus, a total of 85 participants, including 43 in the intervention group and 42 in the comparison group, completed all parts of the study. The mean age of the participants was 77.52 ± 6.15 years. In total, 41.65% of participants were illiterate, and the participants had an average of 1.46 types of chronic diseases. Overall, no substantial difference between the two groups was observed. Table 2 shows the demographic characteristics of the study participants.

### 3.2. Differences in Functional Fitness and Daily Life Activities of the Two Groups

**Figure 2:**
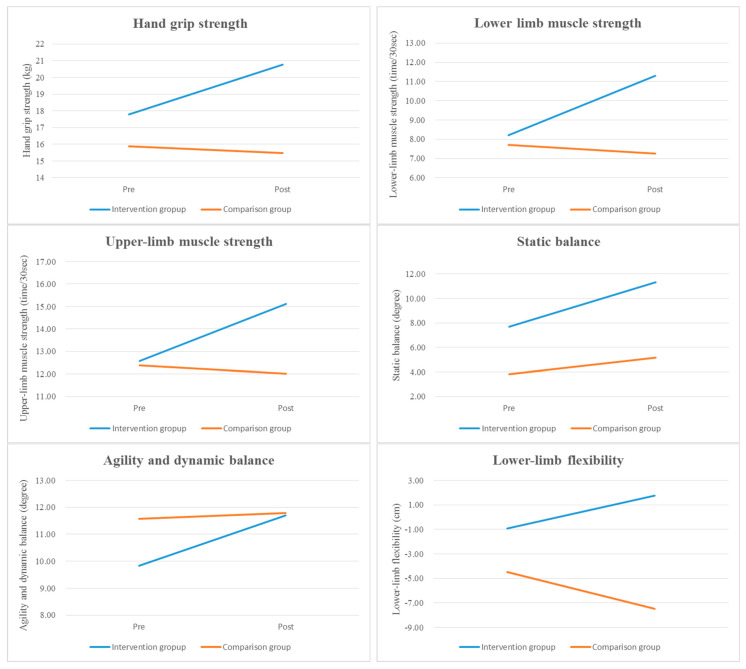

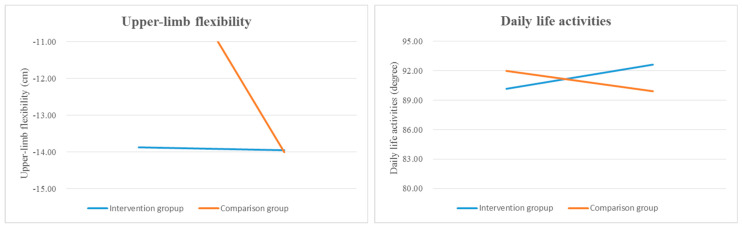
Time−dependent tendencies in functional fitness and daily life activities for the two groups. Note. Pre = pre-test; Post = post-test.

The results of between-group comparisons in functional fitness and daily life activity scores are shown in Table 3 and Figure 2. Hand grip strength, lower- and upper-limb muscle strength, static balance, agility and dynamic balance, lower-limb flexibility, and daily life activities significantly improved in the intervention group when compared with the comparison group at post-test (all *p* < 0.001), and there was a statistically significant difference in the scores of the intervention group (Scheffe’s test result: ① > ②). However, no significant between-group differences were observed for upper-limb flexibility at post-test (*p* = 0.965). Moreover, the chair yoga activity program considerably increased the participants’ daily life activity scores, with the Cohen’s d effect size of the intervention being 3.06.

## 4. Discussion

This study explores the effects of chair yoga therapy interventions on the functional fitness and daily life activities of older female adults with knee osteoarthritis in communities in Taiwan. According to the outcome, this study shows that implementing the chair yoga program regularly for 12 weeks, two times a week, effectively improved functional fitness and daily life activity scores. The chair yoga program involved swinging the arms in natural parabolas, along with repeated supporting movements, resulting in muscle contractions, which improved muscle strength and endurance. Previous studies have shown similar results, namely, that chair yoga exercises increase both upper- and lower-extremity muscle strength [34,35,36]. Park et al. performed a pilot study on a retirement community and found that agility and dynamic balance increased by 10.43% after yoga therapy intervention during a 12-week period, indicating that chair yoga may be a promising intervention to manage functional fitness and improve daily life activities, thereby reducing fall risk for older female adults with knee osteoarthritis [26]. In another study, Niemelä et al. and Kertapati et al. reported significant changes in both knee extension and quadriceps strength [37,38]. Furthermore, Baum et al. reported significant changes in physical performance after 26 weeks of chair yoga exercise [39]. Based on the conclusions of these studies, chair yoga can potentially mitigate the functional fitness problem in older adults with knee osteoarthritis [32,40,41,42]. Moreover, when combining regular breathing with upper- and lower-limb stretching, blood circulation and oxygen uptake can be facilitated, leading to improvements in functional fitness. Therefore, chair yoga enhances muscular strength, body flexibility, and respiratory function. The positive effects we observed for static and dynamic balance, agility, and lower- and upper-limb muscle strength were similar to those reported by Cheung et al. (2017) and Park et al. (2017), indicating that chair yoga can enhance balance and physical function [26,43].

Furthermore, the effect of chair yoga therapy has also been evaluated regarding its improvements in daily life activities among older female adults with knee osteoarthritis, and in the long term, it can prevent falls or the fear of falling as well as their possible complications. It also helps older adults to enhance their muscles and body flexibility, reduce stress, depression, and chronic pain, and enhance overall well-being and quality of life, meaning that such an intervention increases the daily life activity scores in older adults. Overall, our study results suggest that the implementation of a chair yoga program enhances daily life activity scores among older community-dwelling female adults with knee osteoarthritis. Our study results also resonate with the findings of prior research—chair yoga therapy had a positive short-term effect in increasing daily life activity scores [44]. Moreover, the chair yoga program uses stretching and flexion movements to extend the spine, back, and thighs, which can improve function and daily life activity scores. This finding was similar to a previous report suggesting that balneotherapy improved functional limitations and ameliorated the quality of life [45]. Currently, one of the most frequently prescribed nonpharmacological treatments for osteoarthritis diseases is balneotherapy [46], and it can be combined with different treatment modalities, such as massage, exercise, and health education [47].

Chair yoga exercises complement and add value to existing therapy, improving functional fitness. Balance control yoga exercises are taught in clinical practice because yoga actively engages the mind and body. Yoga exercises result in considerable improvements in the balance and daily life activity scores of older female adults. Balance and daily life activity problems for the elderly with knee osteoarthritis have always been a field of concern for comprehensive geriatric care because, often due to associated multi-morbidities, they lead to high-cost intervention and management, which can result in neglect, further deteriorating the functional status of the elderly population. Owing to such concerns, our study proposes chair yoga exercises as an effective means to improve functional fitness problems and daily life activity scores in the elderly through a more culturally acceptable form of exercise. Involving psychosocial, emotional, and physical functions during chair yoga can improve quality of life, which has been one of the prime aims of elderly long-term care amongst geriatricians.

However, this study has several limitations that must be acknowledged. First, because of community limitations in cooperation, this study was quasi-experimental, which may limit the external validity. Randomized controlled trials could be used to measure the effectiveness of the chair yoga intervention. Second, the intervention was implemented in the community; therefore, the results cannot be generalized to other long-term care institutions. Third, the study outcome was measured based on the pre- and post-test results obtained after the twelve-week intervention. Future studies should consider collecting follow-up data and designing a series study to examine the relationship between the intervention frequency and duration of the chair yoga program. Fourth, there were limitations with this study due to the small sample size, and future studies are needed to explore the other potential benefits of different chair yoga programs for older female adults. Fifth, this study did not include male subjects as a sample population. Future studies must develop interventions with multiple modalities and invite community agencies and leaders to cooperate as partners in implementing health-promoting activities to ensure maximum efficacy.

## 5. Conclusions

Our study’s goal was to apply chair yoga therapy for the nonpharmacologic management of knee osteoarthritis in older community-dwelling female adults. Chair yoga therapy is a feasible and effective intervention for older female adults with knee osteoarthritis, with beneficial effects on functional fitness and daily life activity scores. The WHO recommends the adaptation of interventions based on individual physical capacities to improve healthy aging. Thus, interventions with chair yoga therapy can enable older adults with knee osteoarthritis to adopt and practice the therapy at home as part of their daily life, lessening the risk of their disease progressing to disability.

## Figures and Tables

**Table 1:** Content of the chair yoga therapy activities.

Time	Session	Activity Content
15 min	Warm-up activities	Breathing exercise Neck stretch Shoulder stretch Walking on the spot Reaching both arms forward and waving them up and down Reaching both arms forward and extending them to the left and right
50 min	Main course (1)	Seated mountain pose Seated cat–cow pose Seated chandrasana Seated uttanasana Hero pose Down dog with chair
10 min	Break	Break
20 min	Main course (2)	Balance training while standing (a total of six movements, including down dog while standing, lifting the knees while standing, and the tree pose)
15 min	Cooling down and relaxation	Butterfly pose Sitting side-bend pose Seated spinal-twist pose Reclining twist pose Savasana

**Table 2:** Demographic characteristics of study participants in the intervention and comparison groups at baseline.

Variables	Intervention (*n* = 43)				Comparison (*n* = 42)				*t*	*X* ^2^	*p*
	*M*	*SD*	*n*	*%*	*M*	*SD*	*n*	*%*			
Age (years old)	76.37	6.08			78.66	6.18			−1.03		0.306
Education (degree)										4.37	0.225
Illiterate			15	34.9			21	50.0			
Elementary			18	41.9			18	42.9			
Junior high school			7	16.3			0	0			
≥Senior high school			3	7.0			3	7.1			
Number of chronic diseases (number)	1.57	0.93			1.35	1.39			0.51		0.608
DBP (mmHg)	137.02	14.75			137.83	15.69			−0.137		0.889
SBP (mmHg)	72.46	13.26			67.70	10.32			1.01		0.317
HR (bpm)	75.65	10.61			75.10	6.77			0.14		0.880
BMI (kg/m^2^)	24.81	3.11			25.69	3.82			−0.707		0.486
Hand grip strength (kg)	17.80	2.72			15.88	3.40			1.72		0.093
Lower-limb muscle strength (time/30 s)	8.20	2.62			7.70	2.83			0.57		0.554
Upper-limb muscle strength (time/30 s)	12.58	2.82			12.38	2.13			0.21		0.824
Static balance (degree)	7.71	8.26			3.81	3.88			1.43		0.161
Agility and dynamic balance (degree)	9.83	3.20			11.58	4.28			−1.01		0.316
Lower-limb flexibility (cm)	−0.92	10.60			−4.46	11.40			0.90		0.378
Upper-limb flexibility (cm)	−13.87	17.29			−7.40	13.48			−1.16		0.257
Daily life activities (degree)	20.15	4.13			22.01	5.12			0.60		0.11

Note. DBP = diastolic blood pressure; SBP = systolic blood pressure; HR = heart rate; BMI = body mass index.

**Table 3:** Group comparisons in changes in functional fitness and daily life activities.

Variables	Pre-Test	Post-Test	Estimate (β)	SE	Wald χ^2^	*p*	95% CI		Scheffé
	*M ± SD*	*M ± SD*					LL	UL	
Hand grip strength									
①Intervention	17.80 ± 2.72	20.75 ± 3.72							
②Comparison	15.88 ± 3.40	15.47 ± 3.94							
Intercept			15.88	0.75	16.89	<0.001 ***	14.36	21.34	
Pre (I/C) ^a^			−0.76	1.05	−0.73	0.463	−2.82	1.27	
Intervention (post/pre) ^b^			−1.02	0.19	−5.27	<0.001 ***	−1.43	−0.64	
Group difference from pre to post ^c^			2.09	0.31	6.52	<0.001 ***	1.46	2.73	① > ②
Lower-limb muscle strength									
①Intervention	8.20 ± 2.62	11.30 ± 4.33							
②Comparison	7.70 ± 2.83	7.26 ± 2.71							
Intercept			7.70	0.39	2.64	<0.001 ***	6.27	12.78	
Pre (I/C) ^a^			−0.02	0.50	−0.05	0.861	−1.01	0.95	
Intervention (post/pre) ^b^			−0.06	0.15	−0.48	0.001 **	−0.27	0.25	
Group difference from pre to post ^c^			0.19	0.32	0.58	<0.001 ***	0.46	0.93	① > ②
Upper-limb muscle strength									
①Intervention	12.58 ± 2.82	15.12 ± 5.09							
②Comparison	12.38 ± 2.13	12.02 ± 3.45							
Intercept			12.38	0.56	10.25	<0.001 ***	11.43	16.14	
Pre (I/C) ^a^			−0.47	0.25	−1.87	0.482	−0.98	0.04	
Intervention (post/pre) ^b^			−1.28	0.43	−2.98	0.003 **	−2.12	−0.45	
Group difference from pre to post ^c^			2.23	0.48	4.64	<0.001 ***	1.28	3.15	① > ②
Static balance									
①Intervention	7.71 ± 8.26	11.32 ± 9.92							
②Comparison	3.81 ± 3.88	5.17 ± 4.89							
Intercept			3.81	0.87	8.70	<0.001 ***	3.27	12.54	
Pre (I/C) ^a^			−1.14	4.37	−0.26	0.793	−6.71	5.42	
Intervention (post/pre) ^b^			−0.76	0.23	−3.30	0.001 **	−1.21	−0.31	
Group difference from pre to post ^c^			3.87	0.72	5.31	<0.001 ***	2.44	5.29	① > ②
Agility and dynamic balance									
①Intervention	9.83 ± 3.20	11.70 ± 2.25							
②Comparison	11.58 ± 4.28	11.78 ± 4.13							
Intercept			11.58	3.06	0.70	<0.001 ***	8.93	12.86	
Pre (I/C) ^a^			2.15	3.06	0.70	0.825	−3.82	7.14	
Intervention (post/pre) ^b^			−1.62	0.35	−4.38	<0.001 ***	−2.19	0.93	
Group difference from pre to post ^c^			5.32	1.47	3.65	<0.001 ***	2.31	8.24	① > ②
Lower-limb flexibility									
①Intervention	−0.92 ± 10.60	1.75 ± 7.93							
②Comparison	−4.46 ± 11.40	−7.46 ± 10.67							
Intercept			−4.46	3.25	0.80	<0.001 ***	−8.25	2.38	
Pre (I/C) ^a^			−4.24	4.56	−0.99	0.320	−12.28	3.41	
Intervention (post/pre) ^b^			−1.22	0.87	−1.40	0.001 **	−2.91	0.79	
Group difference from pre to post ^c^			6.01	1.62	3.70	<0.001 ***	2.83	9.19	① > ②
Upper-limb flexibility									
①Intervention	−13.87 ± 17.29	−13.95 ± 15.92							
②Comparison	−7.40 ± 13.48	−14.00 ± 16.74							
Intercept			−7.40	0.39	2.65	0.006 **	−15.53	−5.83	
Pre (I/C) ^a^			−0.02	0.50	−0.05	0.961	−1.01	0.96	
Intervention (post/pre) ^b^			−0.03	0.09	−0.26	0.792	−0.18	0.15	
Group difference from pre to post ^c^			−0.01	0.17	−0.04	0.965	−0.33	0.46	
Daily life activities									
①Intervention	90.15 ± 4.13	92.63 ± 0.40							
②Comparison	92.01 ± 5.12	89.92 ± 0.47							
Intercept			92.01	3.84	3.92	<0.001 ***	87.22	94.29	
Pre (I/C) ^a^			−3.02	5.18	−0.57	0.562	−13.15	7.12	
Intervention (post/pre) ^b^			−2.80	1.07	−2.61	<0.001 ***	−4.90	0.71	
Group difference from pre to post^c^			9.62	2.03	4.74	<0.001 ***	5.65	12.62	① > ②

Note. CI = confidence interval; LL = lower limit; UL = upper limit; I = intervention group; C = comparison group; Pre = pre-test; Post = post-test. ^a^ Reference group: Comparison; ^b^ Reference group: Pre-test; ^c^ Reference group: Comparison × Pre-test. ** *p* < 0.01; *** *p* < 0.001.

## Data Availability

The data of the current study are available from the corresponding author upon reasonable request.
